# Supplementary material for: Repeated inversions within a pannier intron drive diversification of intraspecific colour patterns of ladybird beetles
Source: Nat Commun. 2018 Sep 21;9:3843. doi: 10.1038/s41467-018-06116-1 (PMC6155092; doi:10.1038/s41467-018-06116-1)
Supplement: Supplementary file 1 — Supplementary Information [file 41467_2018_6116_MOESM1_ESM.docx]

**Supplementary Information**

**Repeated inversions within a *pannier* intron drive diversification of intraspecific colour patterns of ladybird beetles**

Ando et al.

**Supplementary Table 1 | The 1st small-scale RNAi screening focusing on wing/body wall patterning genes**

| gene name | phenotype in elytra | tested alleles |
| --- | --- | --- |
| *apterous* | wrinkled elytra | *h^Sp^, h^A^* |
| *araucan* | no phenotype | *h, h^C^, h^Sp^, h^A^* |
| *aristaless* | no phenotype | *h* |
| *blistered* | lethal | *h^Sp^, h^A^* |
| *Cubitus interruptus* | pupal lethal | *h^A^* |
| *decapentaplegic* | smaller elytra | *h, h^A^* |
| *Distal-less* | no phenotype | *h, h^C^* |
| *Epidermal growth factor receptor* | lethal | *h^Sp^, h^A^* |
| *pannier* | transformation of black regions to red regions | *h, h^C^, h^Sp^, h^A^* |
| *wingless* | no phenotype | *h, h^Sp^* |

**Supplementary Table 2 | Primers for cloning cDNA fragments**

| Primer name | Target direction | Sequence (5'>3') |
| --- | --- | --- |
| araucan-F | sense | GGITAYTAYCCITAYGAYCC |
| araucan-R | antisense | CCAIGTCATYTTRTTYTCYT |
| aristaless-F | sense | CARYTIGARGARYTIGARAA |
| aristaless-R | antisense | YTCYTGYTTICKCCAYTTIGC |
| apterous-F | sense | ATGMGIACIWSITTYAARCA |
| apterous-R | antisense | AYCATICKICKCCAYTTIGC |
| blistered-F | sense | GGIATHATGAARAARGCITAYGA |
| blistered-R | antisense | TCIGTYTCYTCRAAICCIGTIGC |
| Cubitus interruptus-F | sense | CCITTYAARGCICARTAYATG |
| Cubitus interruptus-R | antisense | RTGIACIGTYTTIACRTGYTT |
| Distal-less-F | sense | ATGMGIAARCCIMGIACIATHTA |
| Distal-less-R | antisense | YTTISWICKICKRTTYTGRAACCA |
| decapentaplegic-F | sense | GTIGGITGGRAYGAYTGGATIGT |
| decapentaplegic-R | antisense | RCAICCRCAICCIACIACIRYCAT |
| Epidermal growth factor receptor-F | sense | GTITWYAARGGIGTITGG |
| Epidermal growth factor receptor-R | antisense | GCIAICCAYTTIATIGG |
| pannier-F | sense | ATIGAYTTYCARTTYGGIGA |
| pannier-R | antisense | GGYTTICKYTTICKIGTYTG |
| wingless-F | sense | TGGGAGGGGGTACAGAACGCACGAG |
| wingless-R* (Nested Universal Primer) | antisense | AAGCAGTGGTAACAACGCAGAGT |
| rp49-F | sense | ACIAARMAITTYATIMGICA |
| rp49-R | Antisense | TGIGCIATYTCISCRCARTA |

H = A + C + T, I = inosine, K = G + T, M = A + C, R = A + G, S = C + G, Y = T + C, W = A + T

*3' RACE product was used for *wingless*

**Supplementary Table 3 | Primers for cloning full-length cDNAs and conserve intronic sequences of *pannier***

| Primer name | Target direction | Sequence (5'>3') |
| --- | --- | --- |
| Ha-pannier-RACE-1 | sense | GGGCAGGGAGTGCGTCAATTGTGGGGCC |
| Ha-pannier-RACE-2 | sense | CCACCCCTCTGTGGAGGAGAGATGGTAC |
| Ha-pannier-RACE-3 | antisense | GCCACAGGCGTTGCACACCGGTTCGCC |
| Ha-pannier-RACE-4 | antisense | GATGCCGTCCTTGCGCATGGCCAGGGG |
| Cs-pannier-RACE-1 | sense | AATTGCGGCACCAGGACGACGACGCTC |
| Cs-pannier-RACE-2 | sense | AAGCTGCACGGCGTCAACAGGCCTCTG |
| Cs-pannier-RACE-3 | antisense | ATCATCATGTTCTGGGCGTA |
| Cs-pannier-RACE-4 | antisense | GTACGGGCTAAGTTCGGATC |
| Cs-pannier-5 | sense | GATCCGAACTTAGCCCGTAC |
| Cs-pannier-6 | antisense | GGGTCTGTTCATCCCATTCATCTTGTGG |
| Ha_pnr_intron_conserved1_F | sense | TCAGCRAATCTTCACATA |
| Ha_pnr_intron_conserved1_R | antisense | CTCCACTCGTTTATCTTAAT |
| Ha_pnr_intron_conserved2_F | sense | AGAGAAAAGAGACAASTTGA |
| Ha_pnr_intron_conserved2_R | antisense | AAAAGTRTTTSCTTCAGG |
| Ha_pnr_intron_conserved3_F | sense | AATGKATTCAAACCYCAGAC |
| Ha_pnr_intron_conserved3_R | antisense | MGRACGCTGAATGAAAGT |

K = G + T, M = A + C, R = A + G, S = C + G, Y = T + C

**Supplementary Table 4 | Primers for direct sequencing of *Ha-pannier* ORF**

| Primer name | Target direction | Sequence (5'>3') | Use of primer |
| --- | --- | --- | --- |
| Ha-pannier-ORFa-F | sense | GCCACTGTCCGTAATTAGCCCGAACAGG | PCR-1 |
| Ha-pannier-ORFa-R | antisense | TCCACCAGAAATAAGGAAATGAGG | PCR-1 |
| Ha-pannier-seq1 | sense | GGGCAGGGAGTGCGTCAATTGTGGGGCC | Sequencing-1 |
| Ha-pannier-seq2 | antisense | GGGTCTGTTCATCCCATTCATCTTGTGG | Sequencing-1 |
| Ha-pannier-seq3 | sense | AAAACAAGGTGGTGGTAGT | Sequencing-1 |
| Ha-pannier-seq4 | antisense | ATAAGGTGACGTCCGTTGGAATCCAGA | Sequencing-1 |
| Ha-pannier-seq5 | sense | CCACAAGATGAATGGGATGAACAGACCC | Sequencing-1 |
| Ha-pannier-seq6 | antisense | TCTTGTCTTGTTTATGTCGT | Sequencing-1 |
| Ha-pannier-5’-F | sense | AGTTCTTCCAAGCCCTCTAAAGTTCAACGAC | PCR-2 |
| Ha-pannier-5’-R | antisense | GGGTCTGTTCATCCCATTCATCTTGTGG | PCR-2 |
| Ha-pannier-seq7 | sense | CATCGTCTTCAGATTAGGTGTAACGACG | Sequencing-2 |
| Ha-pannier-seq8 | antisense | ATAAGGTGACGTCCGTTGGAATCCAGA | Sequencing-2 |
| Ha-pannier-exon3B-F1 | sense | GTTTCCACCAACACCTTC | PCR-3 |
| Ha-pannier-exon3B-R1 | antisense | GTCTGGTTGCAGTTAGTATT | PCR-3 |
| Ha-pannier-seq9 | antisense | GATGCCGTCCTTGCGCATGGCCAGGGG | Sequencing-3 |
| Ha-pannier-exon3B-F2 | sense | ACCTTATGAACATGGATAC | PCR-4 |
| Ha-pannier-exon3B-R2 | antisense | GATATGCTTACTGGTGTCT | PCR-4 |
| Ha-pannier-seq10 | sense | TCTCCCCTCTCCGCCGGTCAGTTCTAC | Sequencing-4 |

**Supplementary Table 5 | Primers for RT-PCR**

| Primer name | Target direction | Sequence (5'>3') |
| --- | --- | --- |
| Ha-pannier-1 | sense | TCGAGCCTGGTGAAGAGCGAACCGGG |
| Ha-pannier-2 | antisense | GGGTCTCCGGACGCAGTATTGATCTC |
| Ha-pannier-3 | sense | GCTCCACCTCCGTAGAAGAC |
| Ha-pannier-4 | antisense | AGCCATCAGTTTGGCAGAAG |
| Cs-pannier-1 | sense | GGCGGTGAACGAGATGACAG |
| Cs-pannier-2 | antisense | GCCATCAGTTTAGCGGACGC |
| Cs-pannier-3 | sense | TGTGGAGGCGGGATGGTACT |
| Cs-pannier-4 | antisense | CTGTTGACGCCGTGCAGCTT |
| Ha-rp49-1 | sense | GCGATCGCTATGGAAAACTC |
| Ha-rp49-2 | antisense | TACGATTTTGCATCAACAGT |
| Cs-rp49-1 | sense | AGTGATCGTTATGGCAAGCT |
| Cs-rp49-2 | antisense | TCTGTTTTGCATCAAAAGGAC |

**Supplementary Table 6 | Primers for dsRNA and riboprobe synthesis (*in vitro* transcription, IVT)**

| Primer name | Target direction | Sequence (5'>3') | Use of primers |
| --- | --- | --- | --- |
| T7-KS | sense | TAATACGACTCACTATAGGGAGACCACTCGAGGTCGACGGTATC | IVT template for dsRNA synthesis |
| T7-SK | antisense | TAATACGACTCACTATAGGGAGACCACCGCTCTAGAACTAGTGGATC | IVT template for dsRNA synthesis |
| Ha-pannier-ORF-F | sense | GTCAGCATGTTCCACACC | ORF cloning |
| Ha-pannier-ORF-R | antisense | TCTTGTCTTGTTTATGTCGT | ORF cloning |
| T3 | sense | ATTAACCCTCACTAAAGGGA | IVT template for riboprobe synthesis |
| SP6-Ha-pnr-NR | antisense | atttaggtgacactatagaACTCCATGGCACTCTC | IVT template for riboprobe synthesis |
| T7 | antisense | TAATACGACTCACTATAGGG | IVT template for riboprobe synthesis |
| SP6-Ha-pnr-CF | sense | atttaggtgacactatagaAAACAAGGTGGTGGTAGT | IVT template for riboprobe synthesis |

**
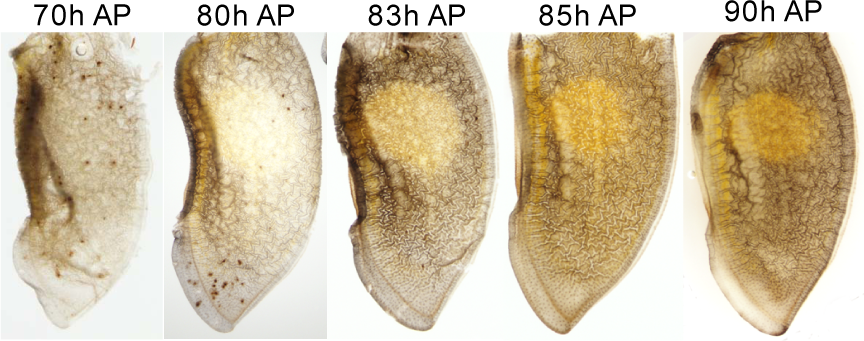
**

**Supplementary Figure 1 | Snapshots of pharate adult elytral pigmentation in *H. axyridis***. Melanin synthesis activity (black) and carotenoids accumulation (orange) were simultaneously visualised using *h^C^* elytra stained with PO activity. Developmental stages are indicated above the images. Strong black and orange signals appeared after 80 h AP.

**
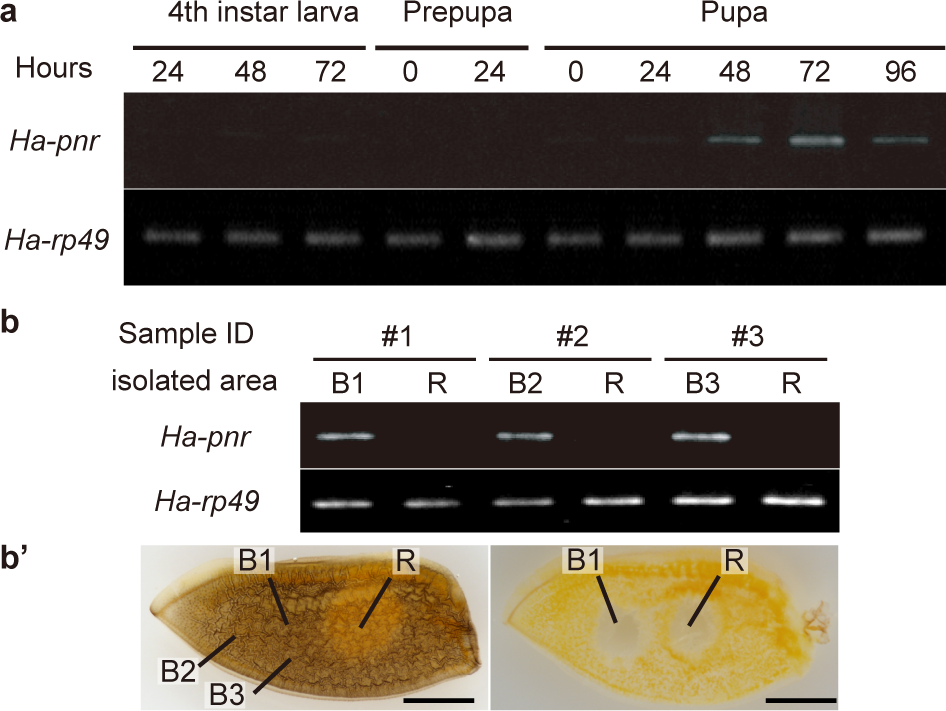
**

**Supplementary Figure 2 | Expression analysis of *H. axyridis* (*Ha*) *pannier* in elytral primordia by RT-PCR. a**, Developmental expression profiles of *pannier* from the final instar larvae to pupae. *Ha-pnr,* *Ha-pannier. Ha-rp49,* *Ha-ribosomal protein 49* (internal control)*.* Adult emergence is at 108 h AP in our rearing condition. Hours after the onset of each stage are indicated above. **(b, b')** Spatial distribution of *pannier* at 84 h AP. Future black (B1-B3) and red (R) regions were isolated for RT-PCR. Pharate adult elytra of three individuals were analysed (#1-#3). *rp49*, internal control. **b',** Left panel, a pharate adult elytron with 3-hour PO staining (84 h AP). Right panel, an example of an elytron after isolation of red and black regions. Scale bars, 1 mm.

**Supplementary Figure 3 | Contiguity of the *pannier*-locus scaffold of Platanus2 for the *H. axyridis* F2-3 sample**. The scaffold was segmented into 2 kbp-windows, and links of 15 kbp-mate-pairs between windows (≧3 mate-pairs) were visualised as arcs. The entire region of the scaffold was uniformly covered by the mate-pairs. No links connecting a distal window-pairs inferring mis-assembly were observed.

**Supplementary Figure 4 | Dot plots of the genomic scaffolds including the *pannier* locus. a−d,** The dot plots between the consensus scaffolds obtained by reassembly of F2-3 mate-pair reads with Platanus2, and each consensus scaffold obtained by *de novo* genome assembly of the 10x linked-reads. **a,** *h^C^* (F2-3) vs. *h^C^* (NT6). **b,** *h^C^* (F2-3) vs. *h^A^* (F2-hybrid). **c,** *h^C^* (F2-3) vs. *h* (NT8). **d,** *h^C^* (F2-3) vs. *C. sep*. The colour code for colouring the homologous segments is on the right side of each panel. Blue arrow, the *pannier* locus. The green and the magenta pins indicate the positions of the breakpoint genotyping markers located on the outermost side, of all genotyping markers found to show association with the *h* locus in the three crossing experiments (*mbl* and *Mink* in Supplementary Table 2, respectively). The subset regions of each scaffold between these two markers or the corresponding regions were extracted for dot plot analyses. In each pair of comparison, sequential linear homology between the two scaffolds was confirmed. The entire responsible region of each *h* allele (*h^C^*, 690 kb; *h^A^*, 660 kb; *h*, 2.1 Mb + α) was included in each single linked-read genomic scaffold (**a**−**c**). In *C. septempunctata*, the genomic region corresponding to the region just downstream of the *pannier* locus in *H. axyridis*, was located in another scaffold different from that including *pannier* (scaffold 47 and 92, respectively), implying at least one translocation event in either of the two ancestral lineages (**d**).

**Supplementary Figure 5 |** **Dot plots of the gap-filled scaffolds around the *pannier* locus.**

**a**-**c**, Dot plots between DNA sequences around the *pannier* locus in different alleles obtained by *de novo* genome assembly of the 10x linked-reads, and following gap-filling using long reads. **a,** *h* (NT8) vs. *h^C^* (NT6). **b,** *h^C^* (NT6) vs. *h^A^* (F2-hybrid). **c,** *h^A^* (F2-hybrid) vs. *h* (NT8). Forward and reverse alignments are depicted as blue and red dots, respectively. The exon-intron structure of *pannier* (1A isoform) in each allele is depicted beside each graph. The red and blue bars beside the exon-intron structures indicate the inversion and translocation positions in the other comparisons, respectively. Traces of inversions (light red squares) were consistently found in every allele comparison within the 1st intron of *pannier*. Combination of inversions (light red squares) and a translocation (a light blue square), presumably formed through successive two inversions, were found in *h*−*h^C^* comparison (**a**).

**Supplementary Figure 6 |** **Polymorphism in ORF sequences of *pannier-A* isoform in the 4 allelic strains of the *h* locus. a,** Nucleotide polymorphisms in *pannier* ORF in 4 allelic strains of *h* (*h*, *h^A^, h^Sp^, h^C^*). R = G or A, Y = T or C, M = A or C, S = G or C, W = A or T. **b**, Amino acid sequences deduced from ORF sequences in (**a**). X = T or S. The alternative exon region (exon 3A) and GATA zinc finger domains are indicated with grey and yellow, respectively.****

**Supplementary Figure 7 |** **Polymorphism in ORF sequences of *pannier-B* isoform in the 4 allelic strains of the *h* locus. a,** Nucleotide polymorphisms in *pannier* ORF in 4 allelic strains of *h* (*h*, *h^A^, h^Sp^, h^C^*). R = G or A, Y = T or C, M = A or C, S = G or C, W = A or T. **b**, Amino acid sequences deduced from ORF sequences in (**a**). X = T or S. The alternative exon region (exon 3B) and GATA zinc finger domains are indicated with grey and yellow, respectively. ****

**Supplementary Figure 8 |** ***vestigial* mRNA is upregulated in the presumptive black regions from early pupal stages in the *h^C^* background.** Read counts of *vestigial* mRNA extracted from the RNA-seq analysis data were plotted. 1B, black region at 24 h AP. 1R, red region at 24 h AP. 3B, black region at 72 h AP. 3R, red region at 72 h AP. Bars, mean read counts. (n = 3). Error bars, standard error of means. *, FDR < 0.01.

**
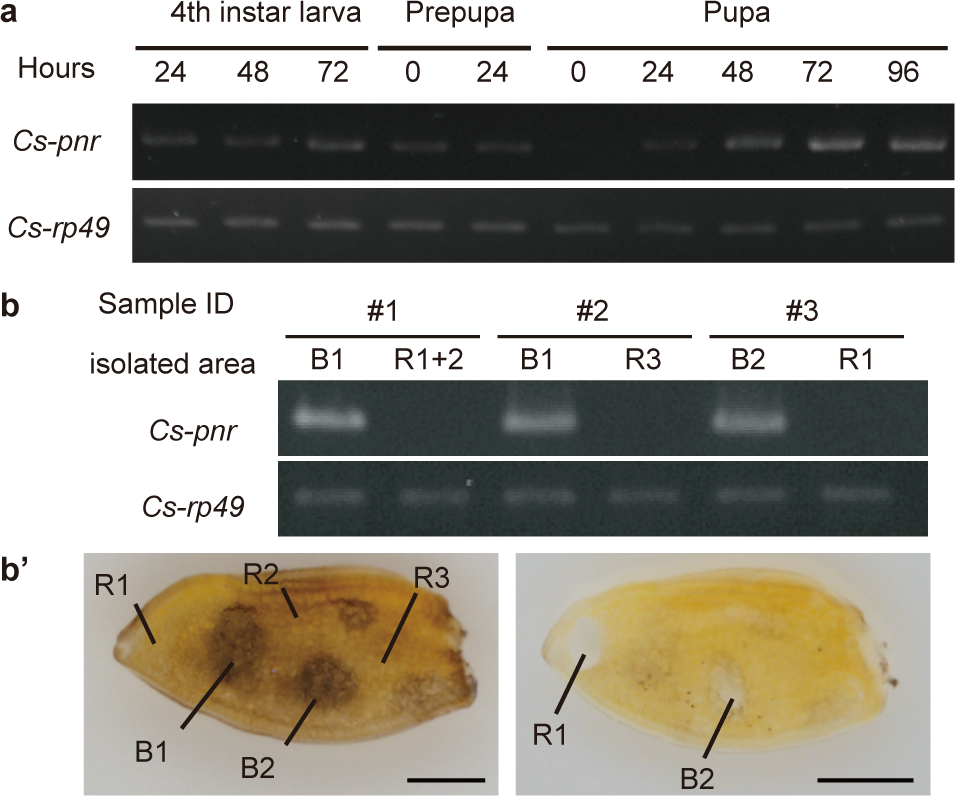
**

**Supplementary Figure 9 | Expression analysis of *C. septempunctata* (*Cs*) *pannier* in elytral primordia by RT-PCR. a**, Developmental expression profiles of *pannier* from the final instar larvae to pupae. *Cs-pnr,* *Cs-pannier. Cs-rp49,* *Cs-ribosomal protein 49* (internal control)*.* Adult emergence is at 108 hours AP in our rearing condition. Hours after the onset of each stage are indicated above. **b, b',** Spatial distribution of *pannier* at 84 h AP. Future black (B1−B2) and red (R1−R3) regions were isolated for RT-PCR. Pharate adult elytra of three individuals were analysed (#1−#3). *Cs-rp49*, internal control. **b',** Left panel, a pharate adult elytron with 3-hour PO staining (84 h AP). Right panel, an example of an elytron after PO staining for 1 hour and isolation of red and black regions. Scale bars, 1 mm.
