# Supplementary material for: Repeated inversions within a pannier intron drive diversification of intraspecific colour patterns of ladybird beetles
Source: Nat Commun. 2018 Sep 21;9:3843. doi: 10.1038/s41467-018-06116-1 (PMC6155092; doi:10.1038/s41467-018-06116-1)
Supplement: Supplementary file 3 — Description of Additional Supplementary Files [file 41467_2018_6116_MOESM3_ESM.pdf]

Description of Additional Supplementary Files

File Name: **Supplementary Data 1**
Description: **Markers and Primers for gene association study on the *h* locus**

File Name: **Supplementary Data 2**
Description: **Statistics of the genome assemblies**

File Name: **Supplementary Data 3**
Description: **Raw count data, statistics and annotations of the RNA-seq analyses in the *h* locus candidate region**

File Name: **Supplementary Data 4**
Description: **Motif enrichment in the upper noncoding regions of pannier in *H.axyridis* and *C. septempunctata***

File Name: **Supplementary Data 5**
Description: **DNA sequences used in the molecular phylogenetic analyses**
a, The aligned nucleotide sequences of *pannier* ORF before trimming gapped and shifted regions. **b,** The aligned nucleotide sequences of *pannier* ORF after trimming. **c,** The aligned nucleotide sequences of the conserved intronic regions of *pannier* (concatenated three blocks) before trimming gapped and shifted regions. **d,** The aligned nucleotide sequences of the conserved intronic regions of pannier after trimming. A dash indicates a gap.

File Name: **Supplementary Data 6**
Description: **Oligo sequences used in the Flexible ddRAD-seq**
